# Interfacial Enrichment of Lauric Acid Assisted by Long-Chain Fatty Acids, Acidity and Salinity at Sea Spray Aerosol Surfaces

**DOI:** 10.1021/acs.jpca.4c03335

**Published:** 2024-08-06

**Authors:** Abigail C. Dommer, Mickey M. Rogers, Kimberly A. Carter-Fenk, Nicholas A. Wauer, Patiemma Rubio, Aakash Davasam, Heather C. Allen, Rommie E. Amaro

**Affiliations:** † Department of Molecular Biology, University of California, San Diego, La Jolla, California 92093, United States; ‡ Department of Chemistry and Biochemistry, The Ohio State University, Columbus, Ohio 43210, United States; § Environmental Molecular Sciences Laboratory, Pacific Northwest National Laboratory, Richland, Washington 99354, United States; ∥ Department of Chemistry and Biochemistry, University of California, San Diego, La Jolla, California 92093, United States

## Abstract

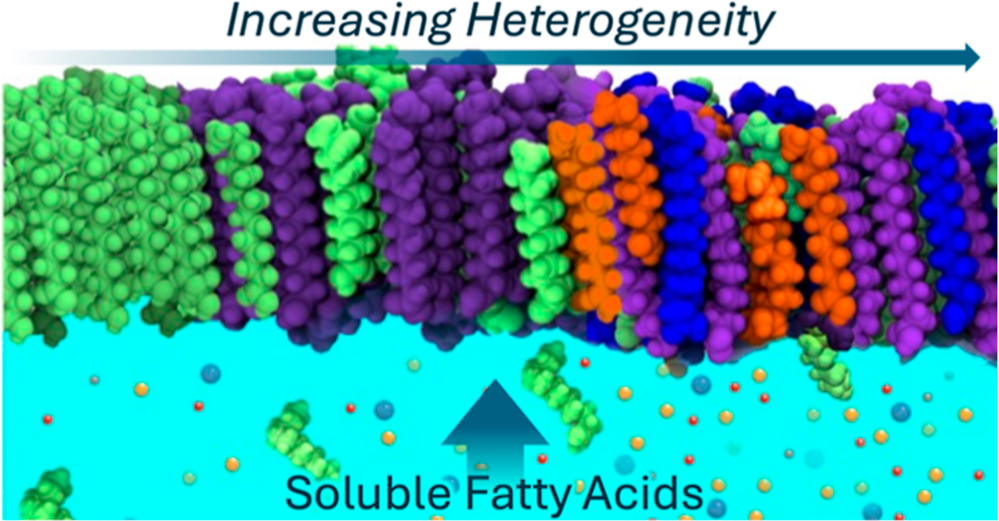

Surfactant monolayers at sea spray aerosol (SSA) surfaces regulate various atmospheric processes including gas transfer, cloud interactions, and radiative properties. Most experimental studies of SSA employ a simplified surfactant mixture of long-chain fatty acids (LCFAs) as a proxy for the sea surface microlayer or SSA surface. However, medium-chain fatty acids (MCFAs) make up nearly 30% of the FA fraction in nascent SSA. Given that LCFA monolayers are easily disrupted upon the introduction of chemical heterogeneity (such as mixed chain lengths), simple FA proxies are unlikely to represent realistic SSA interfaces. Integrating experimental and computational techniques, we characterize the impact that partially soluble MCFAs have on the properties of atmospherically relevant LCFA mixtures. We explore the extent to which the MCFA lauric acid (LA) is surface stabilized by varying acidity, salinity, and monolayer composition. We also discuss the impacts of pH on LCFA-assisted LA retention, where the presence of LCFAs may shift the surface-adsorption equilibria of laurate—the conjugate base—toward higher surface activities. Molecular dynamic simulations suggest a mechanism for the enhanced surface retention of laurate. We conclude that increased FA heterogeneity at SSA surfaces promotes surface activity of soluble FA species, altering monolayer phase behavior and impacting climate-relevant atmospheric processes.

## 1 Introduction

Biogenic organic material accumulates at the sea surface microlayer (SSML) and is ejected into the atmosphere via sea spray aerosol (SSA).^1−5^ Submicron SSAs (<200 nm diameter), which influence the chemistry of the atmosphere and climate through their cloud-forming abilities and radiative properties, are composed primarily of organic material in an approximately 9:1 mass ratio of organics to inorganics, with up to 51% water at 70% relative humidity.^6,7^ Numerous experimental studies have aimed to fully characterize the molecular speciation of the organic fraction, but instrumental and sampling limitations have prevented the identification of more than 25% of the molecules.^2,3,8−13^ Regardless, the major classes of organic molecules found in SSAs, and their relative abundances, have been successfully identified through quantitative bulk analyses integrated with single-particle methods.^13^ Free fatty acids (FAs, C8–C24) have been observed to make up a dominant fraction of the organic material, followed by proteinaceous material (including free amino acids), free saccharides and polysaccharides, and additional organic surfactants such as phospholipids and triacylglycerides.^2,6,13^

Saturated FAs, produced in marine environments by heterotrophic microbiota and phytoplankton, lend distinctive properties to the air/seawater interface. Long-chain FAs (LCFAs) (C ≥ 16), specifically palmitic acid (PA, C16) and stearic acid (SA, C18), are present in up to 75% of the FA mass fraction in fine (dry diameter < 2.5 μm) SSAs.^2^ Extremely surface active, LCFAs are enriched at the SSML, exist as self-assembled monolayers, and are transferred into SSAs via bubble bursting mechanisms at the ocean surface.^14^ LCFAs exhibit unique interfacial properties; namely, their tight hexagonal packing structure leads to crystalline-like monolayers with high melting points, low fluidity, and a nearly complete impermeability to water.^15−18^ Medium-chain FAs (MCFAs, C8–14) are also present in significant amounts (up to ∼30% of the total FA mass fraction), with myristic acid (MA, C14), lauric acid (LA, C12), and nonanoic acid (C9) in highest abundance.^2^ Generally, saturated FA solubility increases with decreasing chain-length; MA is considered partially soluble while LA and other shorter chains show much lower surface activity in experimental measurements of homogeneous monolayers.^19−24^ Due to their partial solubility, MCFA monolayers exist in a metastable state and remain in dynamic equilibrium with their monomers in solution, and predicting their surface retention is complicated based on acid–base equilibria.^20,22,25,26^

The complex monolayers that form at the air/seawater interface help modulate many climate-relevant properties of SSA,^27^ including water evaporation and condensation,^16,17,28^ gas transport and reactive uptake,^29−33^ cloud and ice nucleation,^34,35^ adsorption of polysaccharides and protein,^36−38^ and photochemistry.^39^ Since MCFAs are partially soluble and more challenging to control, they are excluded from most experimental and theoretical studies of proxy marine SSMLs and model SSAs which tend to include only LCFA components. In response, some studies have probed the surface activity of partially soluble MCFAs such as nonanoic acid (C9).^19−21^ Two recent studies in particular have incorporated atmospherically relevant mixtures of saturated FAs into their experimental protocols. Carter-Fenk et al. showed that mixed FA monolayers of MA, PA, and SA at varying pHs exhibit notably different surface properties than their pure FA counterparts.^23^ Most recently, Xu et al. investigated the surface properties of the MA/PA/SA mixture with the addition of mono- and disaccharides to the underlying aqueous solution revealing that hydrogen-bonding (H-bonding) networks are crucial for ocean to atmosphere saccharide transfer.^40,200^

In the present work, we integrate experimental and computational methods to examine the stability and dynamics of mixed chain-length FA monolayers with special attention to the incorporation of soluble surfactants. We show the conditions under which LA can be enhanced at the interface, including in the presence of salt and increased acidity. Building upon previous studies, we also investigate how the incorporation of LCFAs into the MCFA monolayer impacts the monolayer phase behavior and reveal the corresponding role of NaCl. We then use a proxy FA mixture mimicking that in marine aerosol environments to explore how varying pH influences the structure, stability, and dynamics of the resulting interface. Finally, we report on the climate-relevant microphysical properties of heterogeneous mixed FA monolayers to highlight the impacts of chemical complexity at SSA interfaces and the necessity of including MCFAs in laboratory studies of SSA.

## 2 Methods

### 2.1 Experimental Methods

#### 2.1.1 Solution Preparation

SA (≥99%, Sigma), PA (≥99%, Sigma), MA (≥99%, Sigma), LA (99%, Acros Organics), and l-glutamic acid (Sigma) were used without any further purification. Each chemical was dissolved in chloroform (HPLC grade, Fisher Chemical) at a concentration of ∼2 to 3 mM. The concentrations of PA and SA were calibrated by performing surface pressure–area (Π–A) isotherms on water at pH 5.6 and adjusting the concentrations until the lift-off points occurred at 26 and 24 Å^2^/molecule, respectively. The MA concentration was calibrated via a Π–A isotherm on water at pH 2.0 to minimize desorption into the aqueous solution subphase; its concentration was adjusted such that the lift-off point occurred at 55 Å^2^/molecule. LA could not be calibrated with a Π–A isotherm due to its solubility, so its concentration was determined using the mass (Mettler Toledo XS104 Analytical Balance) added to the chloroform. The FA mixtures were prepared using the individual FA solutions described above. All mixtures consisted of molar ratios of their respective components (1 LA: 2 MA: 4 PA: 3 SA, 2 MA: 4 PA: 3 SA, 1 LA: 9 SA, 1 LA: 1 PA, 1 LA: 3 PA, 3 LA: 1 PA). Aliquots of the individual lipid solutions were transferred using a micropipette (FisherBrand Elite).

#### 2.1.2 Surface Pressure–Area (Π–A) Isotherms

Π–A isotherms were measured on a Teflon Langmuir trough (KSV NIMA) with an attached tensiometer and Delrin barriers (KSV NIMA). Both the trough and barriers were thoroughly cleaned with reagent alcohol (Histological grade, Fisher Chemical) and ultrapure water. Surface pressure was measured as a function of mean molecular area (MMA) using either a filter paper plate (Ashless, Whatman), where the paper plate was fully wetted, or a platinum plate (39.240 mm perimeter), which was flamed before running an isotherm. To check for surface cleanliness prior to beginning an experiment, the trough was filled with its aqueous subphase and compressed at the maximum compression speed (270 mm/min/barrier) to check for any significant rise in surface pressure (≤0.20 mN/m). A clean microsyringe (50 or 100 μL, Hamilton) was used to spread the lipid solution dropwise onto the aqueous subphase, and 10 min were allowed for chloroform to evaporate after spreading. The monolayer was symmetrically compressed at a rate of 10 mm/min (5 mm/min per barrier). All isotherms were completed in triplicate and were conducted at 20.4 °C (±1.5 °C).

### 2.2 Computational Methods

#### 2.2.1 Experimental Design

To probe the impacts of adding longer chain FAs to LA monolayers, simulations were performed of FA monolayer mixtures consisting of a 1:0 or 1:1 ratio of protonated LA to protonated PA. Potential of mean force (PMF) simulations were performed in which a single LA molecule was removed from either a pure LA monolayer or a binary LA/PA monolayer at low MMA of 20 Å^2^/molecule in a 0.4 M NaCl solution. To unravel the impacts of surface pressure on molecular rearrangement in binary mixtures, 5 replicates of 100 ns each were carried out over pure water and a salt solution of 0.4 M NaCl at high and low MMAs of 23 and 20 Å^2^/molecule, respectively. The binary mixtures were structurally characterized, and the dynamics were evaluated for indicators of molecular aggregation. To understand the impacts of pH on monolayers of mixed FAs, monolayer simulations were performed using a marine-relevant selection of LA, MA, PA, and SA at a ratio of 1:2:4:3, respectively, each with starting MMA of 20 or 23 Å^2^/molecule over a 0.4 M NaCl solution. Classical MD simulations were run in 5 × 100 ns replicates. All systems are tabulated in Table S1 in Supporting Information.

In order to simulate different pH conditions of mixed FA monolayers using classical MD, we estimated the fraction of protonated to deprotonated FAs based on experimental and theoretical calculations of their surface p*K*_a_s.^19,24^Table 1 gives the protonated to deprotonated residues used for each system. To note: we simulated a pH of 7 instead of pH 5.6 as was used in experiments, given that the numerical values between acid and conjugate base at the microscale are nearly indistinguishable from those at pH 2. At pH 5.6, the acid/base fraction is 69:1 for LA, and ∼190, ∼550, and ∼20,000 for MA, PA, and SA, respectively. These values will make a measurable difference in a macroscale experimental system, but not on an atomic scale where each monolayer contains ∼100 FA molecules. Thus, pH 7 (∼95% protonation) was chosen as an intermediate stage between pH 2 (100% protonated) and pH 8.2 (60% protonated) to enable us to investigate more closely the impacts of varying protonation states within the monolayer.

**Table 1:** Protonated to Deprotonated Fractional Abundances and Approximate Numbers of Residues Used in Each system^a^

acid	p*K*_a_	acid/base	mol acid	mol base
pH 2				
LA	7.44	1:0	20	0
MA	7.88	1:0	40	0
PA	8.34	1:0	80	0
SA	9.89	1:0	60	0
pH 7				
LA	7.44	2.754	16	4
MA	7.88	7.586	36	4
PA	8.34	21.878	76	4
SA	9.89	776.247	60	0
pH 8.2				
LA	7.44	0.173	4	16
MA	7.88	0.479	12	28
PA	8.34	1.380	48	36
SA	9.89	48.978	58	2

a Exact residue quantities vary based on MMA.

#### 2.2.2 Molecular Dynamics Simulations

Explicit solvent all-atom molecular dynamics (MD) simulations were performed using GROMACS^41−43^ version 2020.6 on the Bridges-2 supercomputer^44,45^ at a temperature of 298.15 K with the TIP3P water model^46,47^ and CHARMM36m force fields.^48,49^ The initial configurations for each system were constructed using the CHARMM-GUI Input Generator^50,51^ for monolayers, with an MMA of 20 or 23 Å^2^, approximating an untilted condensed or tilted condensed phase. The water height was set to 45 Å to account for a 12.5 Å cutoff buffer between each monolayer, such that molecules at the surface monolayer or the water box center do not interact with one another other through long-range interactions. The water was neutralized with sodium ions and ionized with 0.4 M NaCl as necessary. The surface area was set to 40 Å to a side for LA and LA/PA systems only, and 45 Å for mixed FA systems. The vertical height for each system was set to 160 Å to allow for enough room to execute steered md and adaptive biasing simulations in which molecules were pulled into and out of the gas phase (vacuum). A visual description of a system setup is provided in the Supporting Information.

#### 2.2.3 PMF Methods

A series of simulations were performed to investigate the free energy changes associated with either pulling an LA molecule from a particular FA monolayer or pulling a water molecule through various quaternary mixtures of FAs. For measuring LA affinity to the FA monolayer, simulations were performed using the steered MD code available in GROMACS 2020.6. An LA molecule whose COM *z*-coordinate was near that of the monolayer COM was selected to be the steered molecule. A harmonic restraint of 1000 kJ mol^–1^ nm^–2^ was applied to the selected molecule and the pulling was executed at a rate of 0.01 nm/ps along the *z* axis, perpendicular to the monolayer plane, over 600 ps. The pull code is provided in the Supporting Information. For each MD frame, the distance and force between the selected molecule and the reference COM (system COM and monolayer COM for water and LA pulling, respectively) were calculated. Molecular configurations along the *z*-axis were extracted every 0.5 Å after the completion of the pulling simulation, and umbrella sampling simulations were launched from each of the 50 starting configurations, using a harmonic restraint between 0.002 and 400 kJ mol^–1^ nm^–2^ as needed. Umbrella sampling was executed for 4 ns at each window in duplicate. The harmonic restraints were adjusted based on the sampling efficiency. To prepare the PMF profiles, the Weighted Histogram Analysis Method (WHAM) was applied via the built-in Gromacs gmx wham analysis tool. More information about this method can be found in the Gromacs manuals.^52^

To measure the impacts of pH on water molecule transport across mixed FA monolayers, the accelerated weight histogram (AWH) method was used as implemented in Gromacs version 2021.5.^53,54^ A water molecule was selected near the bulk aqueous phase COM and sampled across a distance ranging 30 Å above and below the monolayer COM. To maintain a constant MMA, the C1 headgroup atoms were restrained on the *xy* plane with a force constant of 1000 kJ mol^–1^ nm^–2^. The resulting PMF was obtained using the gmx awh analysis tool. The AWH code with all parameters is provided in the Supporting Information, and more information about the method can be found in the Gromacs manuals. Systems were run until successive sampling did not achieve noticeable differences in the PMF profiles. For pH 7, this occurred after full coverage of the sampling region 8 times (10, 21, 46, 118, 226, 474, 1097, and 1769 ns); for pH 2, this was achieved after 4 coverings (120, 194, 577, and 1466 ns).

#### 2.2.4 Additional Analyses

Calculations and analyses reported in this manuscript were carried out using a variety of software and methods, including MDAnalysis,^55,56^ VMD,^57^ GROMACS,^41−43^ PYTRAJ,^58^ and MDTraj.^59^ Molecular visualization and rendering was performed using VMD version 1.9.4a57. Custom Python scripts were written in an iPython Jupyter Notebook environment.^60^ Scripts for constructing graphical networks from MD simulations are provided in the Supporting Information.

## 3 Results and Discussion

### 3.1 Molecular Structure and Stability of Pure LA Monolayers

**Figure 1:**
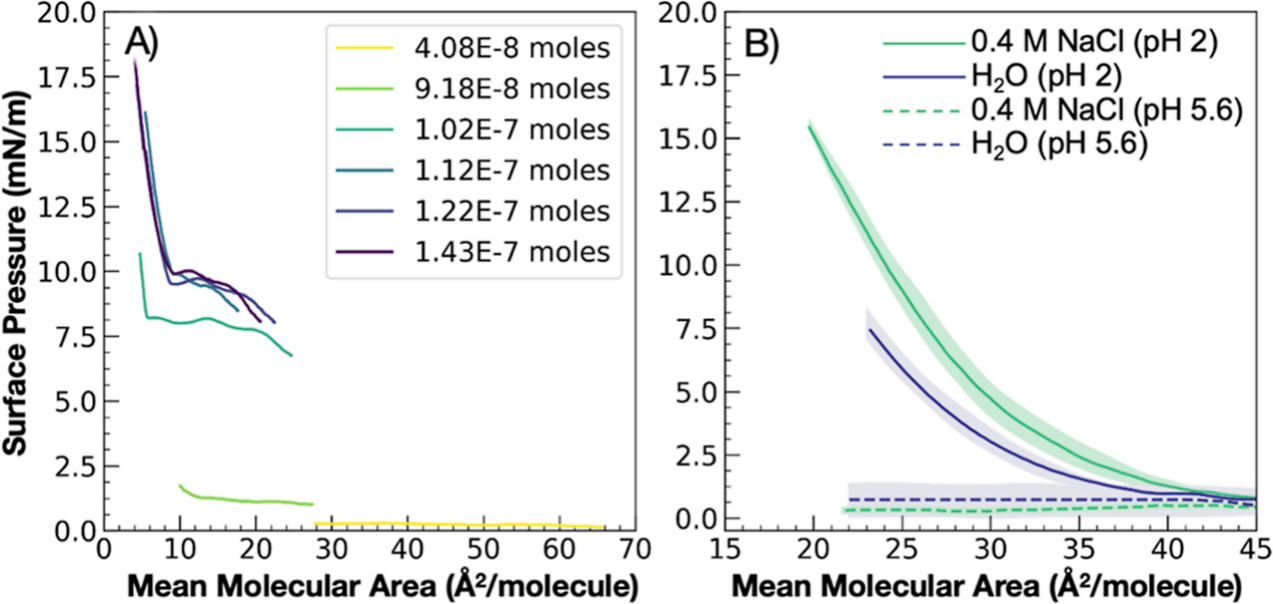
(A) Π–A isotherms showing the concentration dependence of LA surface propensity on ultrapure water at pH 5.6. More moles of LA overcome diffusion-mediated desorption of the acid from the interface. (B) Π–A isotherms showing the relationship between LA surface propensity over atmospherically relevant aqueous solutions: ultrapure water at pH 5.6, 0.4 M NaCl at pH 5.6, ultrapure water at pH 2, and 0.4 M NaCl at pH 2.

MCFAs like LA have been routinely excluded from proxy SSML and SSA systems due to their high solubilities. LA is considered partially soluble such that, though it shows trace surface activity, it is difficult to measure a complete Π–A isotherm due to rapid dissolution of the molecules into solution.^61^ In Figure 1A we show that LA surface activity can be enhanced by increasing the molecular load of the acid at the surface, which enables the surfactant to overcome diffusion-mediated desorption and allows for an observed liftoff.

Decreasing the pH and increasing the NaCl concentration in solution, mimicking the conditions found in nascent SSA (Figure 1B), can also increase LA surface activity. Spreading LA on ultrapure water at pH 2 induces surface activity due to the protonation of the carboxylate headgroups. Π–A isotherms indicate that LA shows a wide liquid expanded (LE) phase for the entire isotherm, a consequence of the decreased solubility at large MMA values. Upon addition of 0.4 M NaCl to the acidified aqueous solution, LA further increases in surface pressure as the Na^+^ confers additional stability to the monolayer.^62^ In contrast, at the same molecular spread at pH 5.6, LA is partially deprotonated and completely desorbs into solution, remaining desorbed even upon the addition of 0.4 M NaCl. This study indicates that pH, rather than salt, is the primary driver of LA to the surface.

The enhanced stability of LA monolayers over 0.4 M NaCl compared to pure water could be due to a combination of factors, including the salting out effect or cation-assisted deprotonation of the carboxylic acid headgroups. The salting-out effect occurs at high enough salt concentrations that water preferentially reorganizes from the solubilized hydrophobic tails to hydrate added ions, leading to the decreased solubility of FAs and increased FA density at the air–water interface.^63−65^ It has also been shown that increasing the salt concentration facilitates headgroup deprotonation even at very low pH, which may increase the stability of the monolayer by increasing ion-dipole interactions between neighboring headgroups.^20,62,66^ Additionally, Na^+^ likely contributes weakly to ion-dipole and solvent-shared ion pairing with fully protonated carboxylic acid groups, though it is unclear to what extent this contributes to the overall stability.

### 3.2 Molecular Structure and Dynamics of Binary LA/PA Monolayers

LCFAs (C ≥ 16) are highly surface active due to their aliphatic chains being sufficiently hydrophobic to prevent desorption into the bulk aqueous phase. In Figure S1, we provide the Π–A isotherm of PA (C16) for comparison to LA. For a pure PA monolayer at pH 5.6, increasing the salt concentration in solution increases the collapse pressure, allowing the monolayer to resist fracture at low MMAs.^67^ However, when both increasing the salt concentration and decreasing pH, we see that pH is again the primary driver of surface activity, as shown by lower pressure (35 mN/m) and an expanded MMA (21.5 Å^2^/molecule) at collapse. These changes in collapse are caused by decreased desorption kinetics induced by the protonation of the headgroup in more acidic conditions. Additionally, salt broadens the collapse structure as it increases the fluidity of the PA monolayer by inducing either electrostatic interactions or intercalation between the carboxylate headgroups, prolonging the eventual collapse.^68^

We can contrast surface enrichment of individual FA monolayers with MCFA and LCFA mixture studies. Π–A isotherms of a 1:1 mixture of LA and PA were performed to measure the potential for LCFAs to assist MCFA retention at the interface. Figure 3 captures the degree to which PA can support LA in a 1:1 mixture with and without the addition of salt.

**Figure 2:**
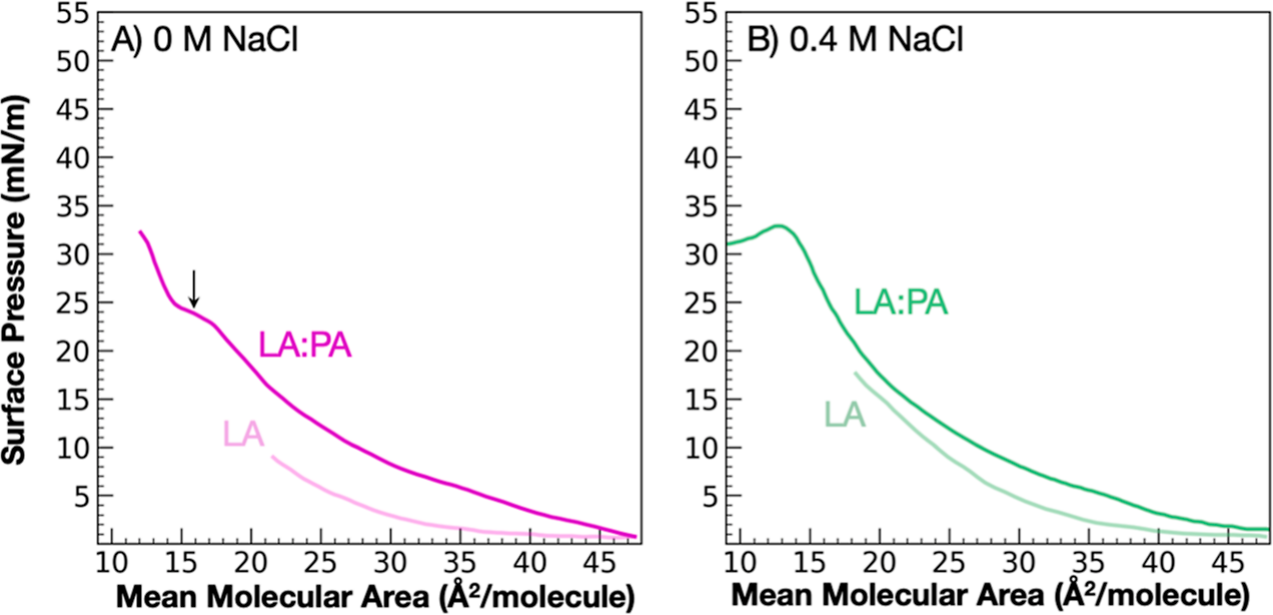
LA/PA mixtures over (A) pure water, pH 2, and (B) 0.4 M NaCl, pH 2, indicating that the addition of PA increases the surface activity of LA under both conditions. The addition of salt stabilizes both FA monolayers, as evidenced by the higher surface pressure values with compression to lower MMA.

**Figure 3:**
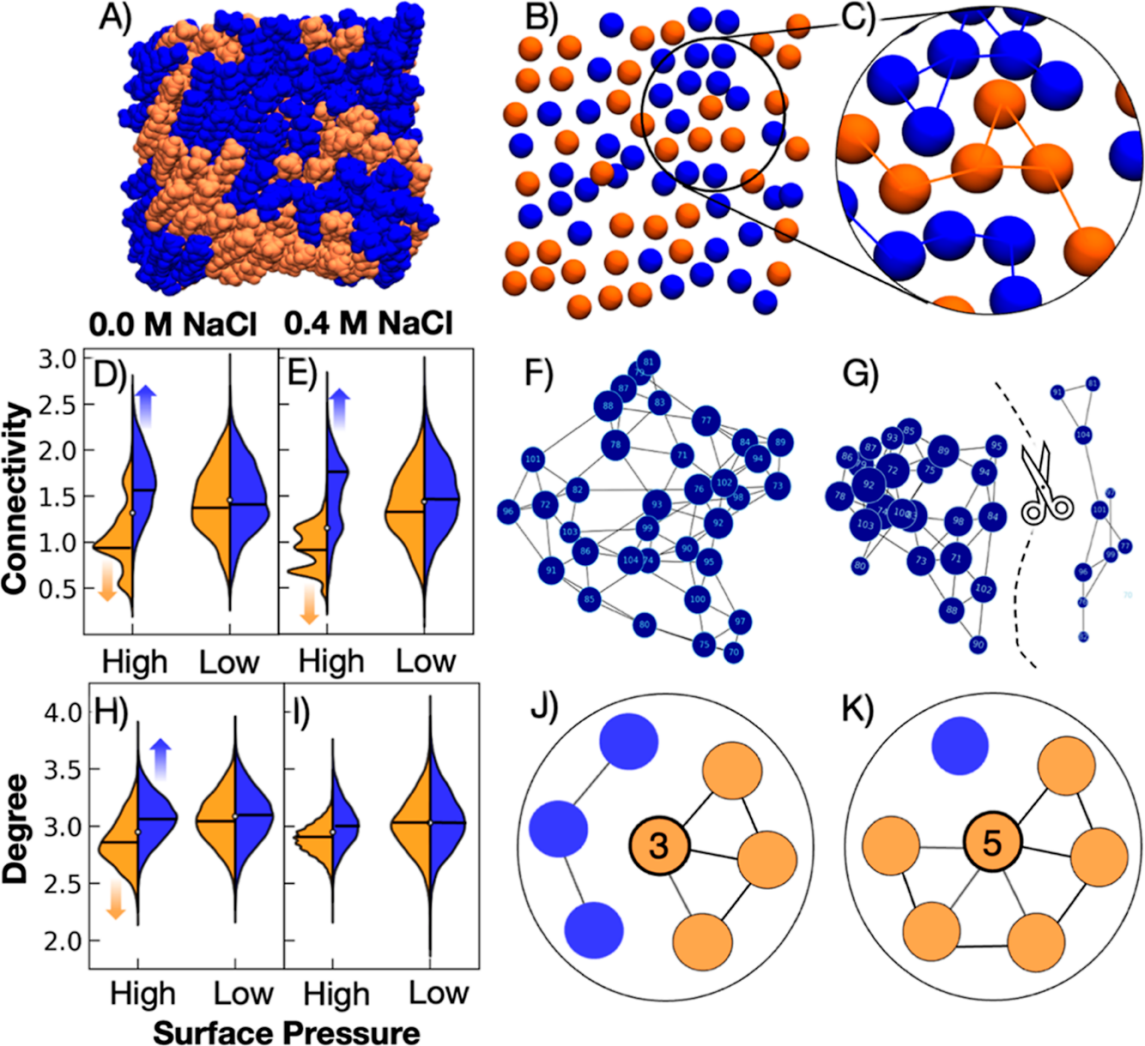
LA and PA aggregation indicators from MD simulations of binary equimolar LA/PA systems, calculated using graphical network analysis. High and low pressures correspond to MMAs of 20 and 23 Å^2^/molecule, respectively. (A) Top-down view of a LA/PA monolayer extracted from MD simulations, where PA and LA are represented in blue and orange VdW representation (VMD). (B) Representative render of C1 headgroups in one monolayer leaflet, with a (C) zoom-in on one region, where connections between same-neighbors are provided. (D,E) Mean connectivity of LA/PA at (D) 0.0 M NaCl and (E) 0.4 M NaCl; average values are provided with a solid black line. (H,I) Mean node degree of LA/PA over (H) 0.0 M NaCl and (I) 0.4 M NaCl; average values are provided with a solid black line. (F) Visual representation of a fully connected network with high connectivity and (G) a disconnected network with low connectivity. (J,K) Schematic diagrams visualizing degrees of (J) 3 and (K) 5, where the central node is bolded, and its degree—number of connected edges—is indicated in the center of the circle. See Supporting Information for additional details and scripts.

In comparing the unary LA and binary LA/PA monolayers spread on aqueous solutions at pH 2, the binary monolayer undergoes an expansion relative to the unary monolayer in both 0 and 0.4 M NaCl conditions. The observed expansion is caused by the increased LA stability when mixed with PA due to enhanced dispersion forces between the FA tails. On pure water (Figure 3A), the binary mixture exhibits a plateau in the isotherm at 17 Å^2^/molecule, indicative of molecular rearrangement upon compression to small MMAs, the exact mechanism of which cannot be determined from the Π–A isotherm alone. In contrast, the LA/PA mixture over 0.4 M NaCl exhibits a full Π–A isotherm (Figure 3B), including post collapse, suggesting that salt stabilizes the binary mixture over the full range of MMAs. Salt-enhanced miscibility has been observed in other mixed monolayer experiments, but the mechanism is still under investigation.^69^

MD simulations can provide additional insights into the monolayer structure and molecular arrangements that might contribute to the experimentally observed isotherms. Here, the CHARMM36 force field is used with the TIP3 water model, a combination known to reproduce experimentally validated biological lipid and protein systems.^48,49,70^ However, it has been well-documented that TIP3 does not accurately predict quantitative measurements of some interfacial properties. For example, surface tension and H-bond relaxation kinetics are more accurately modeled using the more computationally expensive 4-point or polarizable water models.^71,72^ Nevertheless, CHARMM36 and TIP3 can faithfully reproduce relative trends, which, in combination with experimental measurements, offer useful atomistic details of molecular systems.

Analysis of the FA density obtained from molecular simulations of LA/PA mixtures at an MMA of 23 Å^2^ shows that, in the binary mixtures, headgroups of PA sit lower in the monolayer than those of LA (Figure S2), increasing the hydration of PA headgroups and decreasing that of LA. Hydrogen-bonding analysis supports this assessment, with an increase in H-bonds per residue of 4.8 and 6.0% in 0.0 and 0.4 M NaCl solutions, respectively. Over pure water, FA headgroups H-bond to both neighboring headgroups and water molecules in the underlying aqueous phase. However, the addition of salt disrupts H-bonding networks between water molecules while increasing headgroup-water H-bonding and LA–PA H-bonding (Figure S3).^73^ The increase in intramonolayer H-bonding likely contributes to the surface pressure plateau observed in the Π–A isotherms; since LA–PA H-bonding increases stability of the binary mixture, LA is more likely to be retained in the monolayer in the presence of salt and is less easily pushed out at higher surface pressures. In contrast, under no-salt conditions, PA molecules reorganize to maximize dispersion forces, which destabilizes LA at the interface.

Molecular aggregation of PA and LA was evaluated using graphical network theory. Graph theory, typically used in mathematics and data science for ascertaining relationships and transactions between individuals or groups, has recently found useful applications in the topographical analysis of MD simulations. It is possible to use graphical networks to interrogate ion, small molecule, and protein conformations, discover correlated motions,^74−78^ calculate the extent of H-bonding networks,^79^ and elucidate liquid–liquid phase separation dynamics.^80,81^ Here, we use graph theory to identify whether the binary mixtures undergo molecular rearrangement at high surface pressures and under what conditions rearrangement is preferred.

For this analysis, the coordinates of each headgroup carbon were extracted as nodes. To identify linkages between the nodes, nearest same-type neighbors for each carbon headgroup were calculated within a cutoff of 7.2 or 7.4 Å for high- and low-pressure systems, respectively. This distance cutoff was selected based on the radial distribution function between headgroup carbons (Figure S4), at the minimum between the first and second probability density peaks. Neighboring molecules identified by the distance calculation were added to the network and connected with an edge (Figure 3A–C). All frames across 500 ns of each system were analyzed for various indicators of aggregation and connectivity. It is reasonable to hypothesize that some degree of phase separation occurs at low MMAs given the large difference in alkyl chain length between PA and LA, which would explain the plateau in the no-salt isotherm.

The connectivity and node degree of same-type FA networks are plotted in Figure 3, with useful schematics to explain each measurement. Connectivity is a measure of how many edges must be removed to disconnect a node from the entire network, with a higher value indicating that the network is more tightly connected. The degree of a node is the number of edges associated with that node, where a higher node degree indicates more local neighbors. The average connectivity values for high and low surface pressures, respectively, in the presence and absence of 0.4 M NaCl are given in Figure 3D,E. The results show that at higher surface pressures, PA networks exhibit distinctly higher connectivities than LA networks, in both salt and no-salt systems. The low connectivity values associated with LA reflect the expulsion of LA molecules from the interface at high surface pressures, which leads to completely disconnected networks. At lower surface pressures, PA and LA show similar connectivity values, indicating that pressure plays the primary role in aggregating PA molecules together and pushing LA molecules out of the interface.

The values for mean node degree (Figure 3H,I) show that FA molecules are, on average, connected to 3 other same-type neighbors. Since FAs tend to align in a hexagonal packing structure, each with 6 nearest neighbors, this result is expected of an equimolar LA/PA ratio. However, we again see that in high pressure systems, PA molecules tend to have >3 same-type neighbors, while LA tends toward <3 same-type neighbors, strongly indicating that PA molecules experience increased aggregation with other PA molecules. In contrast, LA molecules are pushed into smaller clusters or even removed from the monolayer altogether. The difference in node degree is less apparent in the presence of salt, which is consistent with experiments and H-bond analyses. Salt increases the H-bonding network between molecules at the interface and decreases LA solubility, all of which maintains the miscibility of the mixture and prevents dual collapse.

At high surface pressures, neighboring PA molecules preferentially aggregate given the stronger cohesive forces between their alkyl chains, which may illustrate the molecular rearrangement in the Π–A isotherm observed in Figure 2A. Rather than increase LA–LA aggregation, which would be revealed by similarly increased connectivity and degree values, LA molecules are instead pushed to the periphery of the PA clusters where they undergo desorption from the interface. Thus, although the plateau is reminiscent of a phase coexistence region, complete phase separation is not observed. Phase separation of binary FA mixtures is dependent on the difference in alkyl chain length and is generally not predicted to occur for FAs with differences in chain length less than 6 carbons.^82,83^ These results suggest, however, that the incomplete phase separation of this mixture is due, not to the difference in chain length, but primarily to the solubility of LA, which precludes LA–LA aggregation and leads to dissolution of the molecules from the interface entirely. Surface pressure may similarly facilitate phase separation in binary mixtures of FAs with differences of fewer than 6-carbons, provided that both FAs are insoluble.

### 3.3 LCFA-Assisted Retention of LA in Binary Mixtures

**Figure 4:**
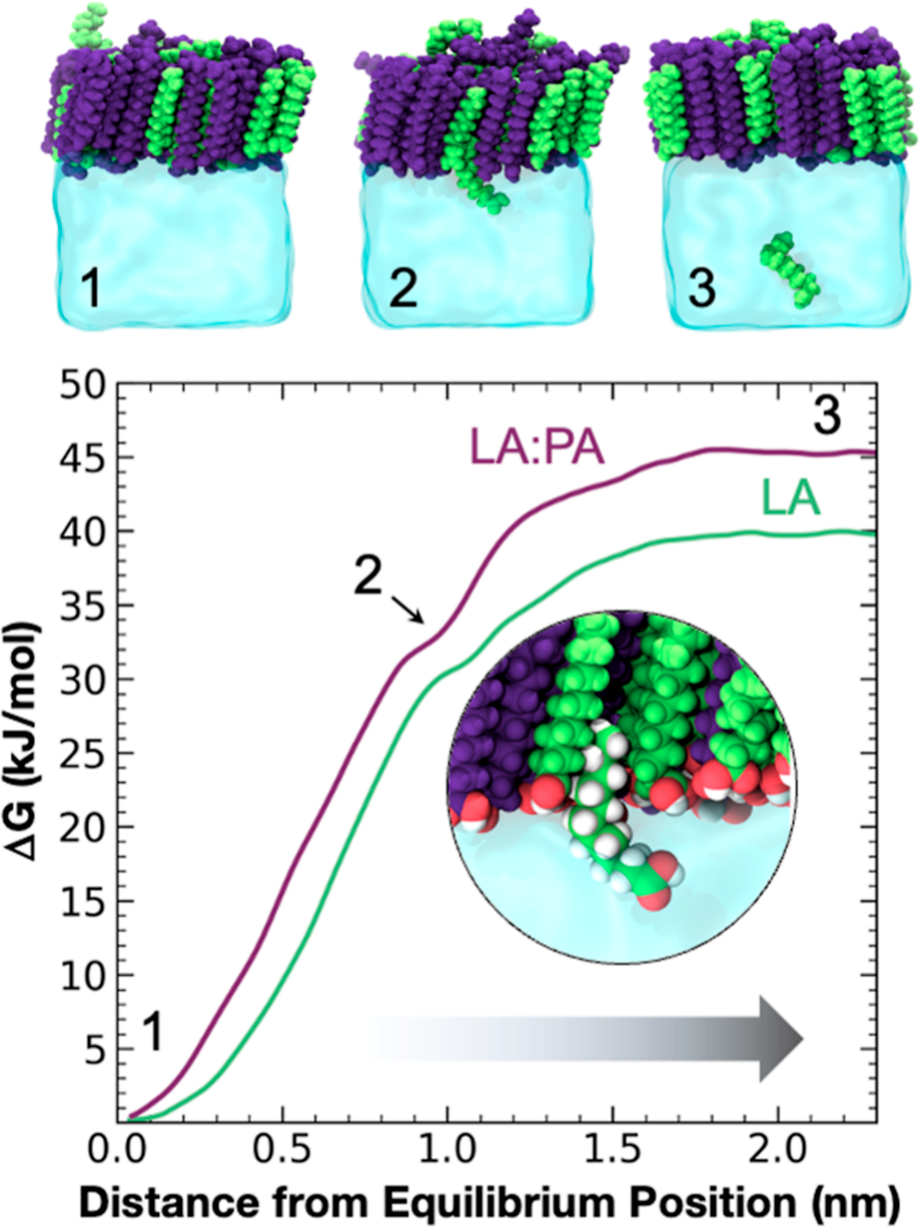
PMF profiles for removing a LA molecule from a homogeneous LA monolayer (green) and from an equimolar binary LA/PA monolayer (purple) at pH 2. Above, snapshots from MD simulations corresponding to distances of the LA molecule from the monolayer center of mass. Snapshots are numerically labeled to correspond to points along the PMF curve.

To computationally quantify the stabilizing effects of LCFAs under SSA conditions, umbrella sampling was used to calculate the PMF associated with pulling an LA molecule out of an FA monolayer into the underlying aqueous phase in the presence and absence of PA. The free energy profiles are given in Figure 4. In both systems, LA molecules begin at their equilibrium positions in their respective monolayers, which represent their lowest energy state; the aqueous solution-facing headgroups participate in hydrogen bonding with surrounding headgroups and/or water molecules, with dispersion interactions between neighboring FAs stabilizing their atmosphere (vacuum)-facing hydrocarbon tails. As the molecule is pulled from the monolayer, the change in free energy (Δ*G*) increases with the increasing exposure of the hydrocarbon tail to the aqueous phase. The slight dip in both of the profiles at approximately 1.0 nm in pulling distance (position 2) corresponds to a conformationally favorable LA position, where both H-bonding interactions and dispersion forces keep the molecule in a bent elbow conformation. This conformation is maintained across ∼1 Å until overcome by the increasing harmonic potential applied by the positional restraints.

The difference in Δ*G* between the two profiles at the 2.0 nm position indicates that it takes more energy to remove an LA molecule from a 1:1 LA/PA monolayer than from a homogeneous LA monolayer at the same MMA. The Δ*G* corresponds roughly to the total contribution of dispersion forces from neighboring FAs at the equilibrium position in the monolayer, which are comprised of 3:3 PA/LA molecules if perfect hexagonal packing is assumed. As the LA/PA ratio changes, Δ*G* will change proportionally to the amount of PA added, increasing with increasing PA and vice versa. For LCFAs to incorporate MCFAs into aggregates in bulk solution, the enthalpic cost of removing aliphatic carbons from neighboring FAs is offset by the entropy gained in freeing up ordered solvation shell water molecules. This has been documented in previous work measuring the cooperative self-assembly of mixed FAs into aggregates such as micelles and bilayers.^84,85^ The assembly of mixed-FA monolayers is likely to be governed by similar thermodynamics principles to bulk-phase aggregates; adding aliphatic carbons to surrounding hydrocarbon tails further stabilizes the LA molecule in the monolayer despite chain-length mismatch.

Given that pH varies as FAs are expelled from the bulk seawater interface to the atmosphere, mixed protonation states add complexity to FA mixtures. The extent to which LCFAs assist MCFA retention in mixed protonation states was explored experimentally by varying the ratio of LA to PA spread on a pH 5.6 aqueous solution. LA is the least surface active in this condition (see Figure 1B), enabling us to highlight the sensitivity of LA surface activity to the presence of PA. Figure S5 shows the Π–A isotherms of a binary LA/PA mixture in the presence and absence of 0.4 M NaCl. These isotherms indicate that PA contributes to the surface-stabilization of LA in both salt and no-salt conditions. Comparing a 3:1 and 1:3 mixture of LA to PA on ultrapure water at pH 5.6, the 3:1 mixture exhibits a more compressed Π–A isotherm compared to the 1:3 mixture (Figure S6). Since the 1:3 LA/PA mixture Π–A isotherm is more abundant in insoluble LCFA, the liftoff begins immediately upon spreading, contrasting the 26 Å^2^/molecule liftoff observed for PA on ultrapure water at pH 5.6. LA promotes disorder and less tight packing for the PA, therefore increasing monolayer fluidity. Given the vastly different isotherms observed, we can conclude that even a small fraction of LCFAs can stabilize MCFA monolayers, and conversely, a small fraction of MCFAs can significantly fluidize LCFA monolayers.

### 3.4 Impacts of LA on SSA Monolayer Proxies

**Figure 5:**
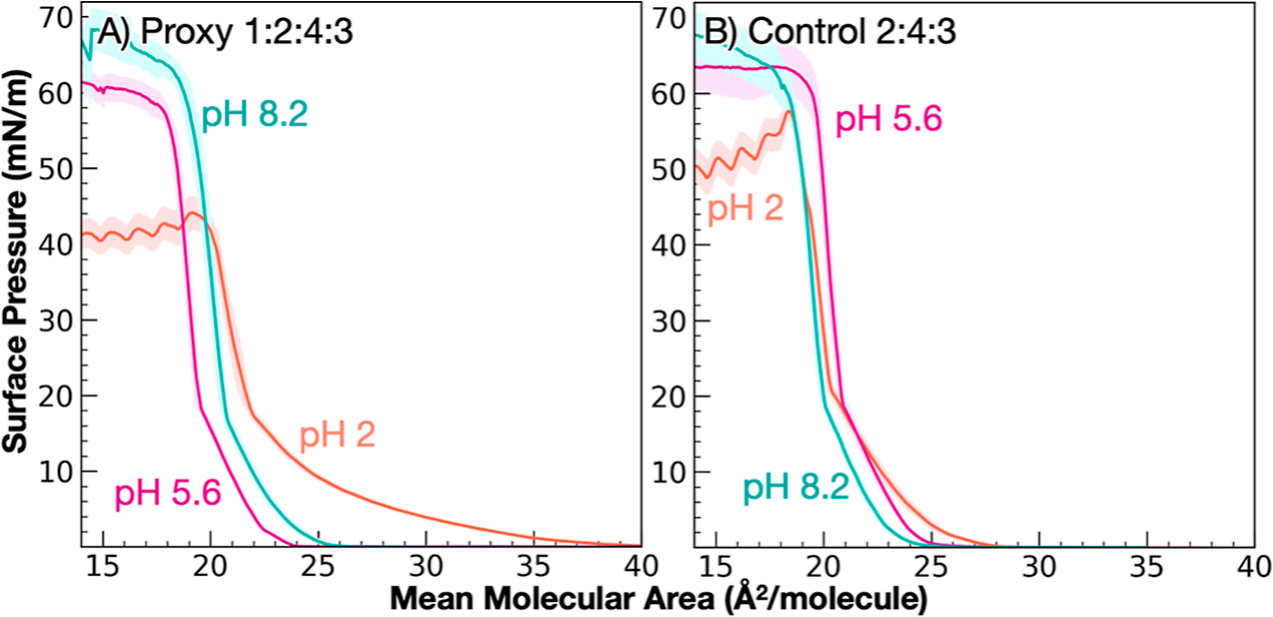
Surface pressure–area isotherms of mixed FA monolayers at pH 2 (orange), 5.6 (magenta), and 8.2 (cyan) on an aqueous solution of 0.4 M NaCl with mole ratios of LA, MA, PA, and SA. (A) Proxy mixture, ratio 1 LA/2 MA/4 PA/3SA; (B) control mixture, ratio 2 MA/4PA/3SA.

We have shown that LA can be surface stabilized in acidic and saline conditions (Section 3.1), as well as in the presence of LCFAs in salt and no salt conditions (Section 3.2). We have also shown even a small fraction of LCFAs can stabilize LA monolayers, including in higher pHs (Section 3.3). It is unclear, however, to what extent LA impacts more complex and varied SSA environments. FAs in real seawater have marked chain-length heterogeneity driven by marine biological processes. Additionally, the pH fluctuates as SSAs are driven from the ocean surface into the atmosphere, dropping rapidly from that of bulk seawater (pH 8.2) to aged aerosol (pH 2) upon interaction with acidic atmospheric gases.^86^ To investigate the impacts of LA on complex, marine-relevant FA mixtures, Π–A isotherms of a quaternary mixture (proxy) are compared to those of a ternary mixture (control) without LA (Figure 5). In this case, the proxy contains LA (C12), MA (C14), PA (C16), and SA (C18), in a 1:2:4:3 mol ratio, respectively, which reflects the ratios of FAs in the highest abundance in submicrometer SSA.^2,13^ The control contains the same mole ratios of MA, PA, and SA with the exclusion of LA.

At pH 2, all FAs are protonated, and LA is expected to have its highest surface activity. Compared to the control, the proxy monolayer shows a higher MMA at lift-off, a lower collapse pressure, and a much more gradual slope in the LE phase from MMA 23–36 Å^2^/molecule. LA is not only surface active in the quaternary mixture, but it also gives the monolayer significantly greater fluidity and compressibility. The disruption to monolayer structure is attributed to the decreased sum of dispersion interactions caused by chain-length mismatch, which leads to monolayer expansion as FAs pack together less tightly.

At pH 5.6, the profiles between the proxy and control mixtures appear fairly similar, with the exception that the proxy mixture shows a slightly lower collapse pressure than the control. The difference between the control mixture and the proxy can be considered in terms of protonation states. The proxy is 93.2% protonated compared to 95.4% of the control due to the differences in overall p*K*_a_ as governed by the individual FA fractions. It is possible that the modest increase in deprotonated headgroups and the destabilizing contribution of the shorter FA tails leads to an earlier collapse as LA is expelled from the surface.

At pH 8.2, the approximate pH of seawater, the profiles of the Proxy and Control isotherms appear similar, suggesting that adding LA has little effect on monolayer phase behavior. Both monolayers exhibit high surface activity and are able to tolerate very high collapse pressures. Previous studies overwhelmingly indicate that FA monolayers are stabilized at equimolar ratios of protonated to deprotonated headgroups (observed by increased lifetimes of FA-based foams and soaps^87−93^). Considered in terms of protonation states, the proxy and control mixtures are 60 and 65% protonated. It is possible that at this pH, the high stability associated with ion-dipole bonding makes the monolayer properties less sensitive to perturbations by trace amounts of MCFAs.

Overall, the high and low pH conditions maintain similar trends across the proxy and control systems, with the more insoluble, low pH condition lifting off at a higher MMA compared to the more soluble, high pH condition. The low pH condition also collapses at a much lower surface pressure compared to the high pH system. However, there are clear differences in liftoff, condensed phases, and collapse pressure when comparing the same pH across the proxy and control systems.

### 3.5 Estimating LA Retention in SSA Monolayer Proxies

At high pH, where the deprotonated form of the FA is more abundant, it is difficult to estimate the overall surface retention of partially soluble FAs. The varying dissociation constants of carboxylic acids at the surface and in the bulk increasingly complicate equilibrium models even without the added stabilizing effect of LCFAs. If ideal mixing is assumed, analytical methods can be employed to estimate the overall retention from Π–A isotherms. However, quantifying % LA retention for the present systems reveals that these FA mixtures exhibit broad deviations from ideal mixing behavior. That is, increasing the mole ratio of deprotonated headgroups leads first to a compression of the mixture as a result of favorable ion-dipole H-bonding, followed by a monolayer expansion due to electrostatic repulsions (see Supporting Information for calculation details). At neutral and basic pH conditions, where pH ≥ p*K*_a_, the headgroups exist in equimolar ratios and undergo ion-dipole H-bonding. Not only does this type of interaction lead to increased stability, but it also leads to a compression effect, as neighboring headgroups can be pulled closer together. At pH 5.6, the proxy system appears to be compressed compared to the control due to this effect. As pH increases, interactions between deprotonated headgroups induce electrostatic repulsions, which lead to an expanded monolayer. At pH 8.2, it is possible that electrostatic repulsion is more dominant, leading to a positive estimate of LA retention. Figure S7 provides an illustration of these interactions.

**Figure 6:**
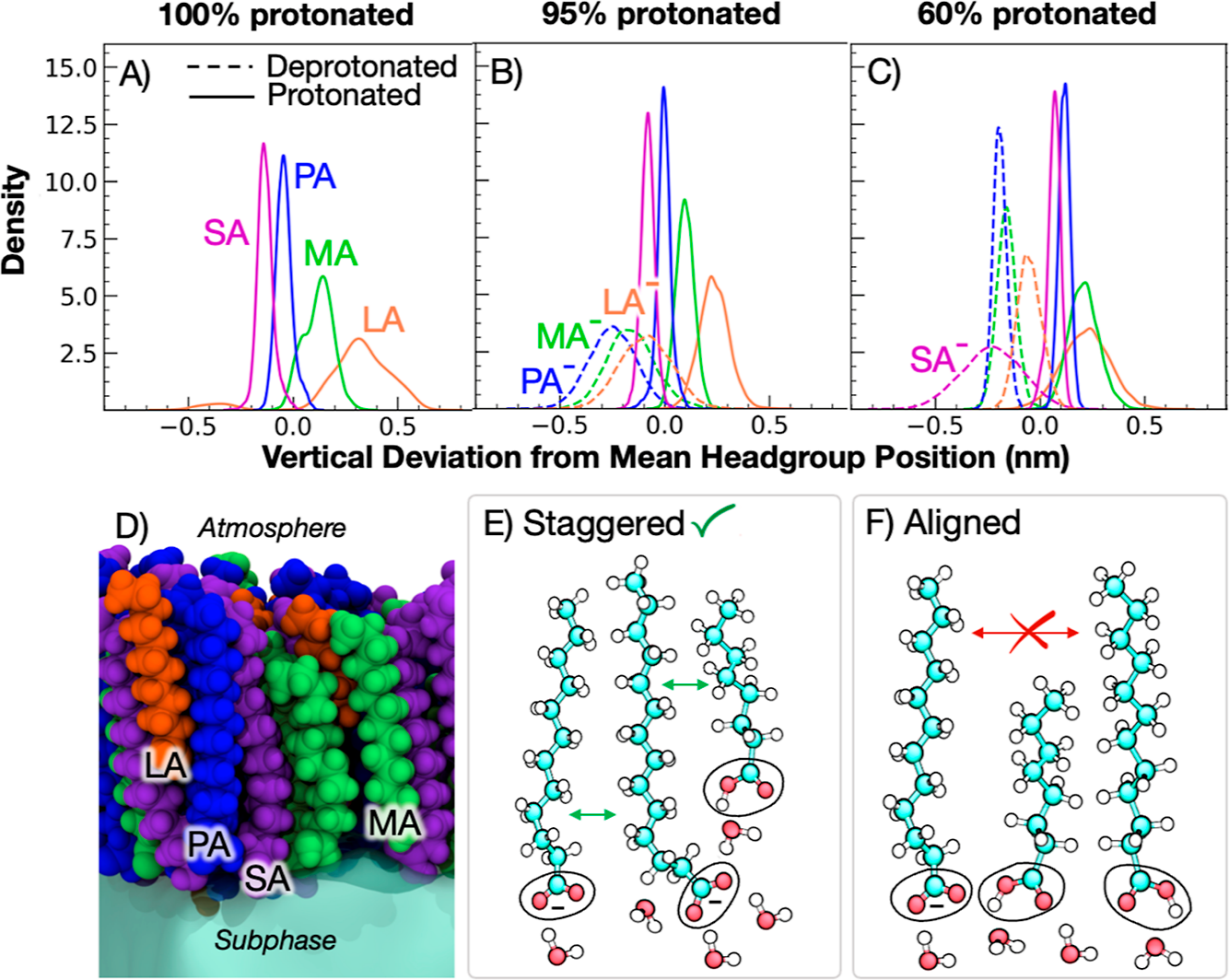
Structure of staggered FA headgroups in mixed FA monolayers. Top row: vertical deviations of headgroup C1 carbons from mean headgroup height at (A) pH 2; (B) pH 7; and (C) pH 8.2. Straight and dashed lines indicate protonated and deprotonated headgroups, respectively. Bottom row: (D) side view snapshot from MD simulations of mixed monolayer at pH 2 showing staggered FAs; (E) schematic of staggered headgroup heights with varying chain lengths; (F) schematic of aligned headgroups with varying chain lengths. Staggered headgroups maximize van der Waals forces of hydrocarbon tails and polar headgroup interactions with neighboring headgroups and water. H-bonding networks form vertical scaffolds throughout the monolayer, promoting surface stability of MCFAs. Aligned headgroups with chain-length mismatch leads to reduced interactions between tails.

Given the difficulty in estimating the relative stability of LA and its conjugate base using experimental methods, MD simulations were employed to gather insights into the structure and dynamics of LA-LCFA mixtures with varying protonation states. These simulations suggest that both the acid and conjugate base of LA are easily retained at high pH due to increased structural stability induced by the surrounding LCFAs. pH impacts the positioning of the FAs in the monolayer, leading to vertical staggering based on both chain length and protonation state. Figure 6 shows the trends associated with chain length heterogeneity and pH.

Figure 6A–C shows the vertical deviation of the C1 headgroup carbons from mean headgroup position for each of the three pH systems. More negative values along the *x*-axis indicate increased hydration with respect to the mean headgroup position. As expected, deprotonated headgroups (dashed lines) are more hydrated than their protonated counterparts. Additionally, as FA chain length decreases, the corresponding FA headgroups become less hydrated. This trend has been observed previously for free FAs embedded in phospholipid bilayers and is attributed to the energy balance between counteracting forces:^94^ the penalty for burying a charged or polar headgroup into a hydrophobic environment is balanced by the hydrophobic interactions gained with increasing acyl chain length. However, in the case of monolayers at the air–aqueous interface, an additional energetic penalty is associated with exposing hydrophobic acyl chains to vacuum (Figure 6F). Thus, the monolayers adopt a structural configuration in which the headgroups are staggered to maximize hydrophobic interactions between alkyl carbons (Figure 6D,E). This configuration also facilitates the formation of H-bonds between neighboring carboxylic acid headgroups and supports greater exposure of polar headgroups to water molecules (Figure S8).

An important consequence of this may be that the conjugate base of soluble FAs is more stable at the interface than estimates would otherwise suggest. The energetic penalty of retaining the anion in the monolayer is relatively lower due to staggered headgroup positioning, as the carboxylate can participate in ion-dipole bonding with neighboring headgroups, and the hydrophobic interactions between neighboring hydrocarbon tails are maximized. The higher chain length heterogeneity and increased diversity in protonation states promotes this added stability, as the headgroups form more vertically scaffolded H-bonding networks throughout the monolayer. These networks are then maintained by the high salt conditions in SSA, which enhance the miscibility of the mixtures.

The addition of MCFAs to LCFA mixtures at high pH has little apparent effect on monolayer physical properties, but the effect of MCFAs increases as pH is lowered. It is unclear to what extent this is due to the increased solubility of LA at high pH, and further study using more surface-sensitive approaches is needed to thoroughly quantify these trends. However, as demonstrated here and elsewhere, both high salt concentration^20,95^ and cooperative self-assembly^85,88,89,93,96^ with LCFAs can enhance surface activity of MCFAs and their conjugate bases.

### 3.6 Climate-Relevant Implications

Surfactant monolayer phase behavior at aerosol surfaces affects water evaporation, condensation, and small molecule adsorption and desorption kinetics.^27,97^ These physical properties in turn influence the phase and reactivity of aerosol particles, the distribution of greenhouse gases^29,98^ cloud formation,^28,99^ and ice nucleation,^34,35^ all of which impact the planetary energy balance. Water evaporation in particular is highly sensitive to surface film structure and rigidity, as influenced by composition, thickness (chain length), surface pressure, and functional groups.^16,17,100−102^

**Figure 7:**
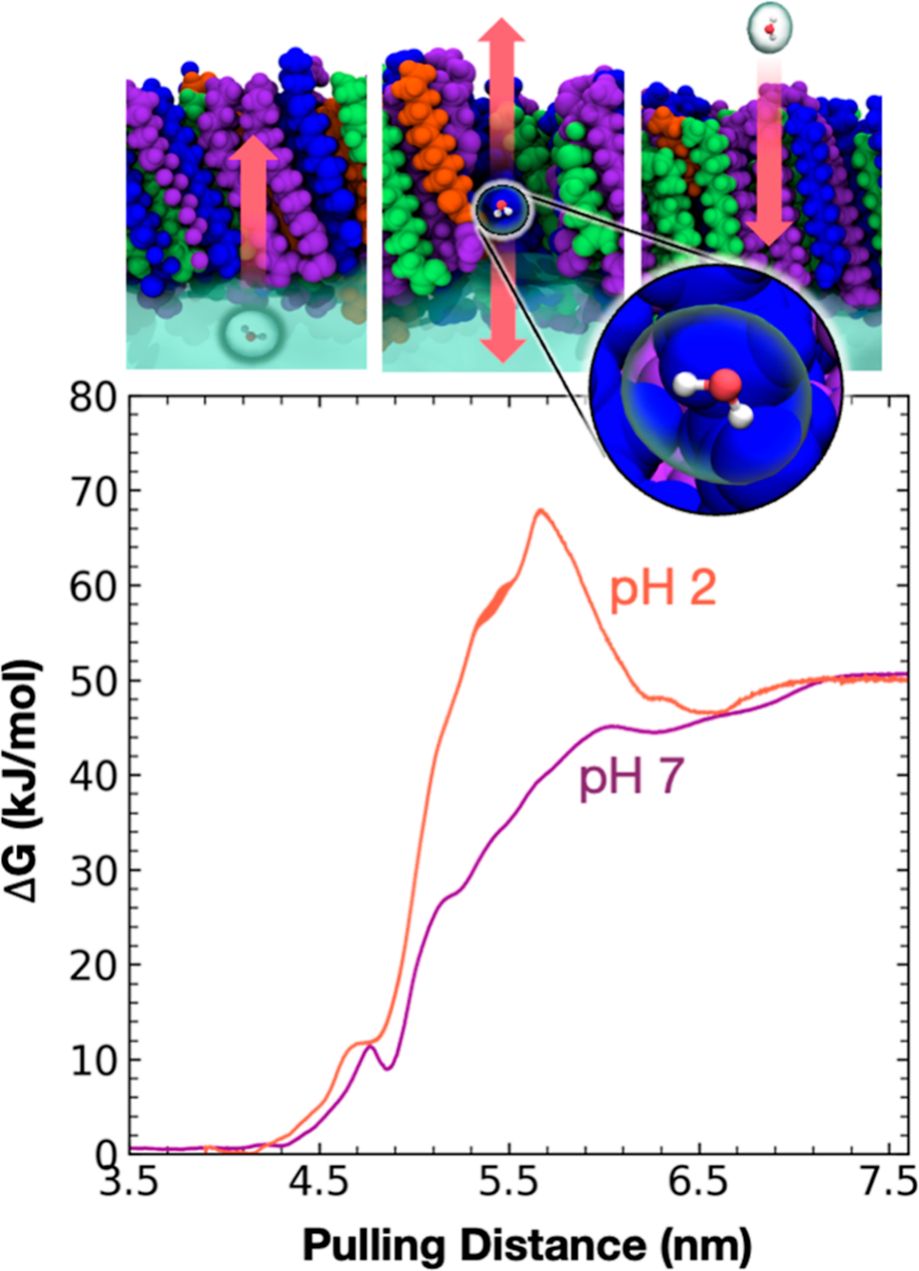
PMF profiles from MD simulations of FA monolayers at pH 2 (SSA pH) and pH 7. All simulations were performed at 20 Å^2^/molecule over a 0.4 M NaCl solution. Headgroups were restrained at their equilibrated positions in the *z* direction; they otherwise had freedom of lateral movement. Water molecules were also restrained with a flat-bottom potential to prevent evaporation with the water molecule being pulled.

We explored the impacts of surfactant heterogeneity on water transport by measuring the PMF of pulling a water molecule through the proxy SSA monolayer in steadily decreasing pH conditions, representing sequential steps in the SSA aging process. Our results are provided in Figure 7. The total Δ*G* difference between the initial and final states corresponds to the solvation energy of a TIP3 water molecule in 0.4 M NaCl; this is a constant value across both simulations. The variation between systems lies in the thermodynamic path required to move from the aqueous phase to the gas phase. The water molecule experiences different interactions as it transits through the monolayer based on FA protonation state, monolayer thickness, and molecular organization.

The PMFs for each pH condition have peaks and valleys that correspond to functional group positions and densities in the monolayers. At low pH, the water molecule experiences a high energy barrier to evaporation once it passes through the carboxylic acid headgroups toward the hydrocarbon tails, where it experiences unfavorable hydrophobic interactions that hinder its movement across the surface. At neutral pH, the headgroup staggering as described above enables the entrainment of water throughout much of the monolayer thickness, seen by multiple plateaus and valleys in the PMF profile. This staggering facilitates the movement of water across the interface, which may have significant effects on water evaporation kinetics. Taken together, this suggests that water evaporation in acidic SSA may be slowed compared to higher pHs despite the increased fluidity of the monolayer due to the presence of MCFAs.

By refining the representation of aerosol dynamics in models, researchers can better assess the complex interplay between aerosols and atmospheric processes. This improved modeling aids in comprehending regional climate variations, precipitation patterns, and the distribution of greenhouse gases, facilitating more informed climate change mitigation and adaptation strategies for a sustainable future. The inclusion of mixed FA monolayers contributes to a more nuanced comprehension of aerosol–climate interactions and enhances climate prediction capabilities.

## 4 Conclusion

Soluble organics require consideration within SSA proxy FA mixtures due to their influence on interfacial film behavior. Here, Π–A isotherms were used to measure monolayer phase behavior and MD simulations were used to investigate the structural and dynamic characteristics of FA mixtures. As the underlying aqueous phase complexity is increased, greater stabilizing effects are observed, supporting the presence of MCFAs at the interface of the SSML and SSA. The MCFA LA is surface-stabilized by salt and is readily incorporated into mixtures of LCFAs, increasing monolayer fluidity and compressibility. MD simulations reveal that the presence of salt enhances the miscibility of FA mixtures and eliminates the characteristic double collapse seen in binary mixtures of FAs. We show that atmospherically relevant proxy monolayer mixtures exhibit the highest sensitivity to MCFA perturbation at low pHs. Additionally, we suggest that LCFAs may aid in the retention of the more soluble conjugate base at higher pHs and provide a mechanism by which the equilibria can be shifted such that the carboxylate form of the FA is more surface stabilized than estimates might suggest. As a result, LA has a large impact on molecular packing at the air/seawater interface despite constituting a small mole fraction of the mixture. Our study highlights the importance of selecting appropriate surfactants for SSML and SSA proxies while lending molecular-level insights into the physicochemical properties of aerosol surfaces that impact their climate-relevant properties. The incorporation of soluble FAs like LA in climate model parameters is essential due to their variable but significant influence on molecular organization at the air/seawater interface.

## Supporting Information Available

Additional figures and tables describing MD simulations, all input scripts used for classical and enhanced sampling simulations, python scripts and workflows for graphical network analysis, and calculation details (PDF)
