# Supplementary material for: Interfacial Enrichment of Lauric Acid Assisted by Long-Chain Fatty Acids, Acidity and Salinity at Sea Spray Aerosol Surfaces
Source: J Phys Chem A. 2024 Aug 6;128(34):7195–207. doi: 10.1021/acs.jpca.4c03335 (PMC11372753; doi:10.1021/acs.jpca.4c03335)

Supporting Information for:

Interfacial Enrichment of Lauric Acid Assisted by Long-Chain Fatty Acids, Acidity and Salinity at Sea Spray Aerosol Surfaces

Abigail C. Dommer,[1†] Mickey M. Rogers,[2,3†] Kimberly A. Carter-Fenk,[2] Nicholas A. Wauer,[4] Patiemma Rubio,[4] Aakash Davasam,[1,4] Heather C. Allen,[2] and Rommie E. Amaro[1]**

*Corresponding Authors: Rommie E. Amaro (ramaro@ucsd.edu) and Heather C. Allen (allen.697@osu.edu)

[†]These authors contributed equally to this work

[1]Department of Molecular Biology, University of California, San Diego, La Jolla, CA, 92093, United States

[2]Department of Chemistry and Biochemistry, The Ohio State University, Columbus, OH, 43210, United States

[3]Environmental Molecular Sciences Laboratory, Pacific Northwest National Laboratory, Richland, WA, 99354, United States

[4]Department of Chemistry and Biochemistry, University of California, San Diego, La Jolla, CA, 92093, United States

Table of Contents

1 Tables

Table S1. Complete list of MD systems S2

2 Figures

Figure S1. Π-A isotherms of pure palmitic acid........ S2
Figure S2. LA:PA MD system diagram at pH 2........ S3
Figure S3. LA:PA H-bond analysis from MD simulations........ S3
Figure S4. Π-A isotherms of LA:PA equimolar ratios at pH 5.6........ S4
Figure S5. Radial distribution functions for high and low pressures LA:PA systems S4
Figure S6. Π-A isotherms of LA:PA mixed ratios at pH 5.6........ S5
Figure S7. H-bonding schematic for FAs with mixed protonation states........ S5
Figure S8. H-bonding probability densities for mixed protonation states........ S6

3 Scripts

Script S1. Gromacs *.mdp code for classical MD simulations S7
Script S2. Gromacs *.mdp code for steered MD sampling simulations S8
Script S3. Gromacs *.mdp code for AWH simulations S8
Script S4. Workflow and code for creating NetworkX graph for network analysis S9

4 Calculations

Calculation 1. LA retention calculations S9

1 Tables

Table S1. Complete list of systems studied with molecular dynamics. The abbreviations UC and TC stand for untilted condensed and tilted condensed phases, respectively.

LA:PA					
**System**	**XYZ**	**MMA**	**Phase**	**Salt**	**Simulation Time**
LA:PA	40 x 40 x 45	20 & 23	UC/TC	0 & 0.4 M NaCl	5 x 100 ns
**LA:MA:PA:SA**					
**System**	**XYZ**	**MMA**	**Phase**	**Salt**	**Simulation Time**
pH 2	45x45x45	20 & 23	UC/TC	0.4 M NaCl	5 x 100 ns
pH 7	45x45x45	20 & 23	UC/TC	0.4 M NaCl	5 x 100 ns
pH 8.2	45x45x45	20 & 23	UC/TC	0.4 M NaCl	5 x 100 ns
**Umbrella sampling**					
**System**	**pH**	**MMA**	**Phase**	**Salt**	**Simulation Time**
LA:PA	2	20	UC	0.4 M NaCl	100 x 8 ns
LA	2	20	UC	0.4 M NaCl	100 x 8 ns
**AWH**					
**System**	**pH**	**MMA**	**Phase**	**Salt**	**Simulation Time**
LA:MA:PA:SA	2	20	UC	0.4 M NaCl	1.5 μs
LA:MA:PA:SA	7	20	UC	0.4 M NaCl	1.3 μs

2 Figures

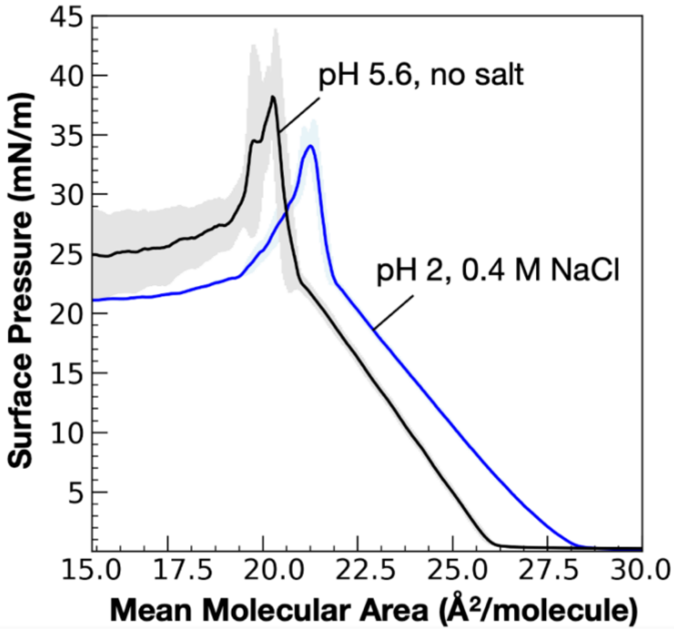

Figure S1. Π-A isotherm of palmitic acid (PA) over pure water at pH 5.6 (black) and 0.4M NaCl at pH 2 (blue).

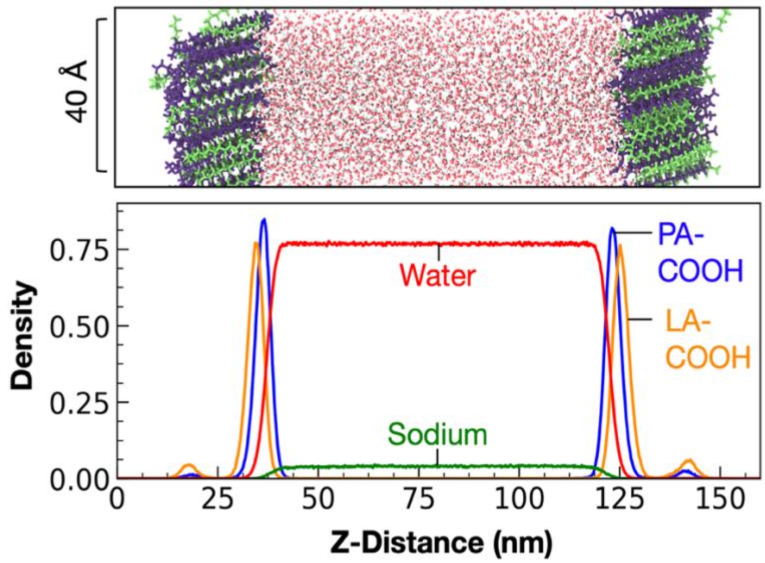

Figure S2. Visual description of protonated binary lauric acid (LA, green licorice): palmitic acid (PA, purple licorice) monolayer molecular dynamics system in the presence of 0.4 M NaCl. Top: Snapshot from molecular dynamics simulation of protonated LA over a 0.4 M subphase. Bottom: Density profile of water (red), sodium (green), LA carboxylic acid headgroups (orange), and PA carboxylic acid headgroups (blue). This figure indicates that the headgroups of the FA stagger in such a way that PA headgroups are more solvated than LA headgroups.

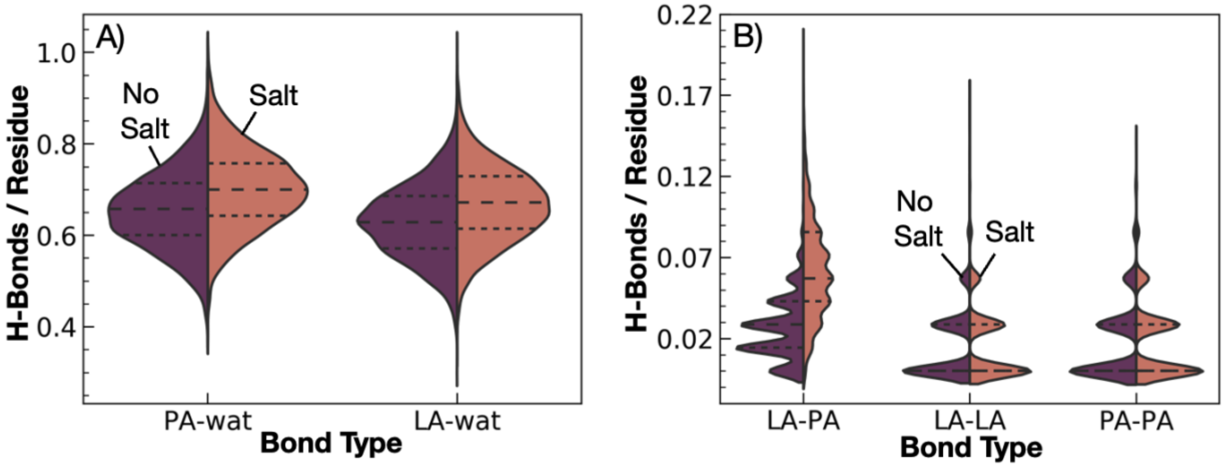

Figure S3. Split violin plots representing H-bonds per residue for LA:PA monolayers at 23 Å^2/molecule over 0.4 M NaCl (coral) and pure water (purple) subphases. H-bonds were calculated using the H-Bonds analysis tool in VMD v1.9.4a57 with a donor-acceptor distance of 2.9 Å and an angle cut-off of 20 degrees.

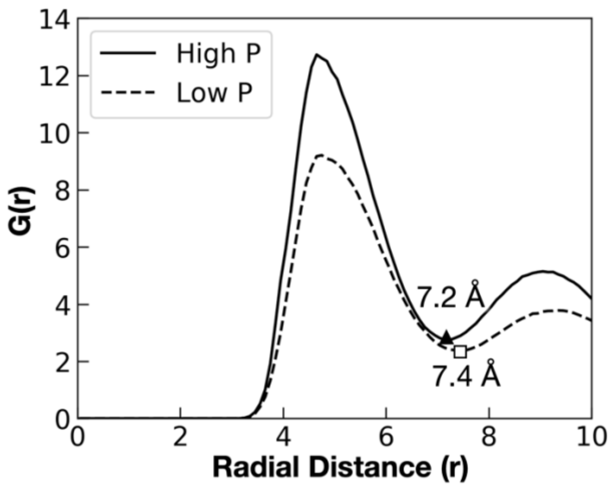

Figure S4. Radial distribution functions, G(r), between C1 headgroup carbons at low pressure (23 $Å^2$/molecule, dashed line) and high pressure (20 $Å^2$/molecule, solid line). The distance cutoffs used to determine connected molecules is given by the minimum between the first and second density peaks are labeled.

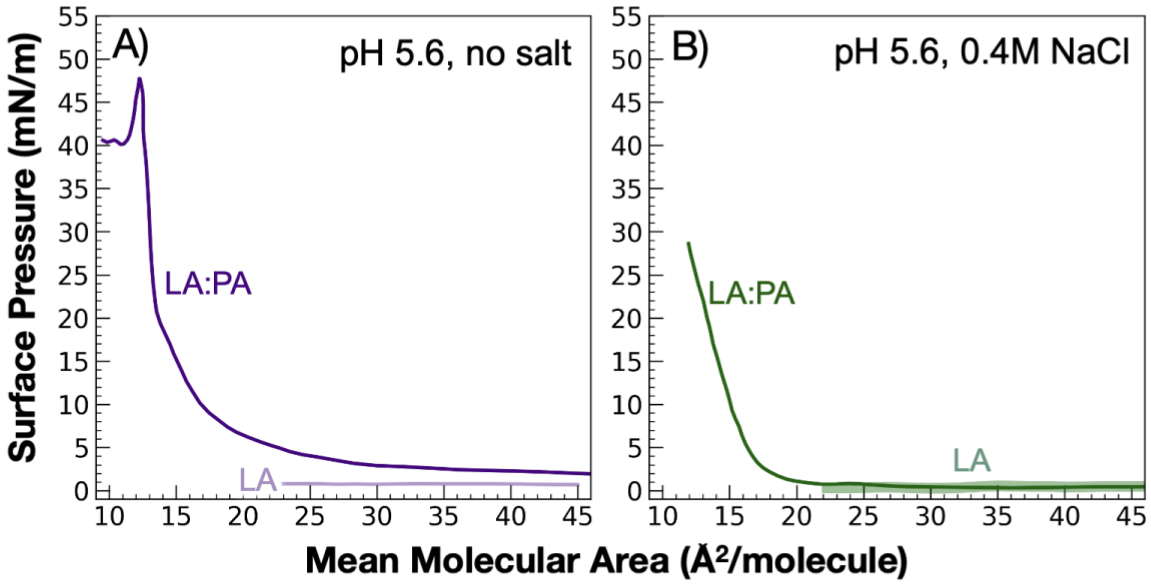

Figure S5. Equimolar LA:PA isotherms at pH 5.6 over a pure water (A) and 0.4 M NaCl (B) aqueous subphase. The profiles, in comparison to PA over pure water, indicate that LA is more surface-stabilized by PA over a salt water subphase than over pure water. The profile of the binary mixture in (A) has significant PA character, with LA contributing to increased compressibility from MMA 15-45 $Å^2$/molecule. In contrast, over salt, the profile less closely matches pure PA as the LA is stabilized by the salt and the mixture remains miscible across a broader MMA range.

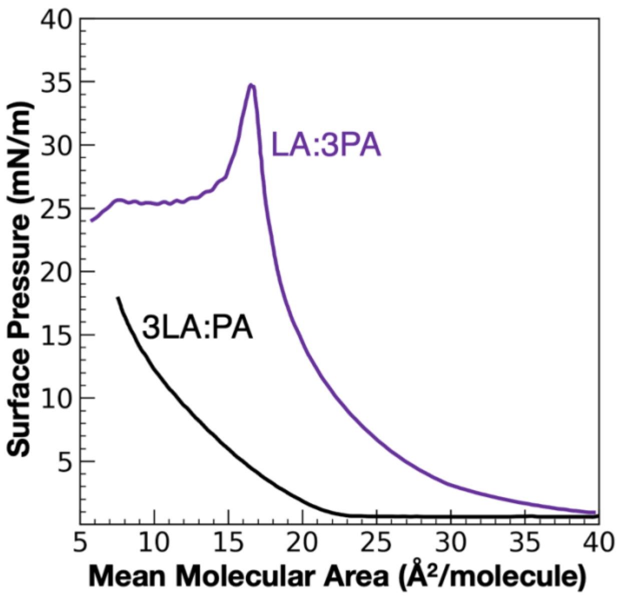

Figure S6. Surface pressure – area isotherms of binary mixtures of lauric acid (LA) and palmitic acid (PA) in a 3:1 molar ratio (black trace) and a 1:3 molar ratio (purple trace). The fatty acid mixtures are spread on pure water at pH 5.6. Greater LA molar contributions lead to a more fluidized monolayer, and increasing the PA molar contribution causes the monolayer to become more rigid due to greater dispersion forces between the long-chain fatty acids.

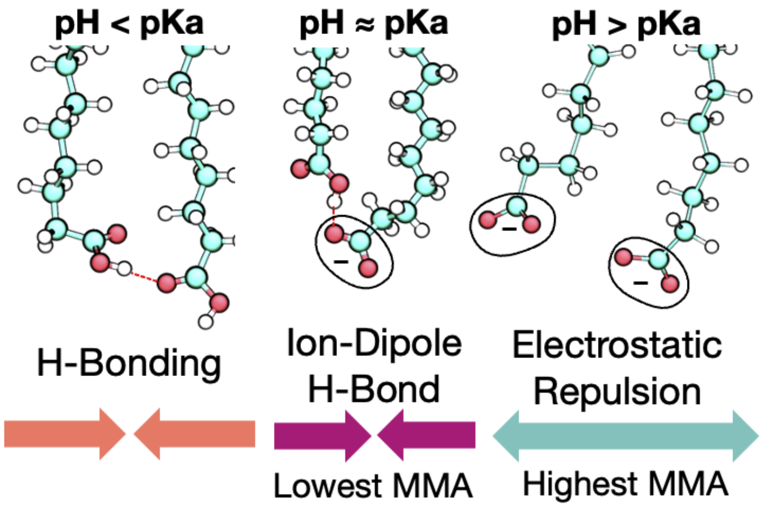

Figure S7. Schematic representing the H-bonding interactions influencing mixing behavior between FAs with mixed protonation states.

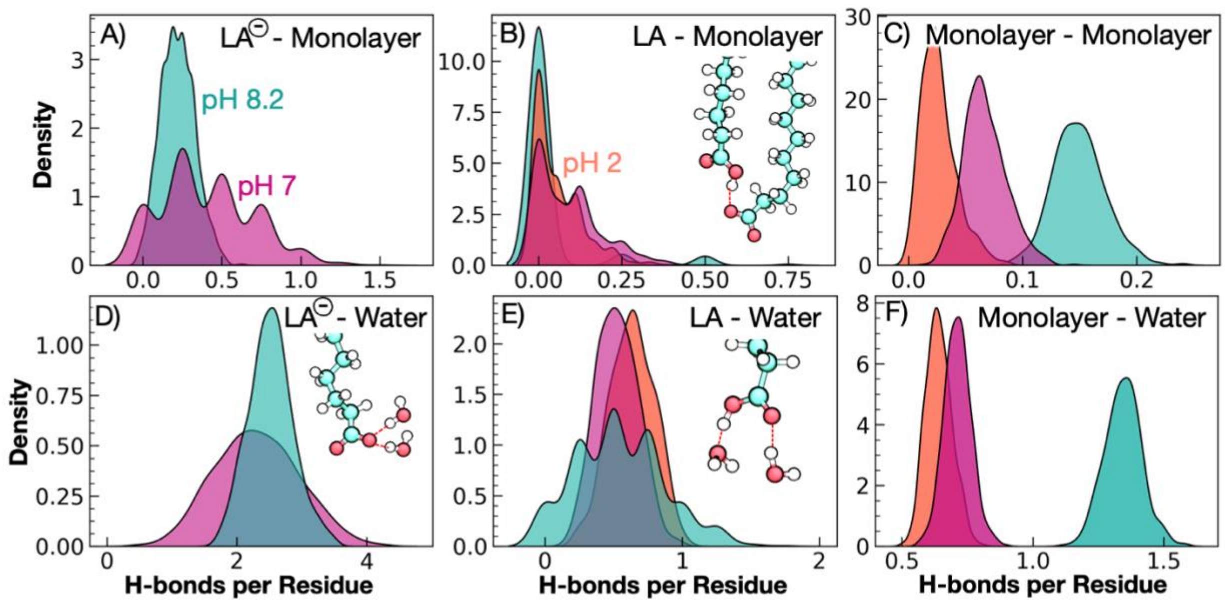

Figure S8. Hydrogen bonding densities for LA-LCFA mixtures at varying protonation states. Each plot corresponds to H-bonds per residue between: A) Deprotonated LA-Monolayer; B) Protonated LA – Monolayer; C) Monolayer – Monolayer; D) Deprotonated LA – Water; E) Protonated LA – Water; and F) Monolayer-Monolayer. Colors correspond to each protonation state: pH 2 (coral), pH 7 (magenta) and pH 8.2 (cyan).

3 Scripts

```
; Classical MD code
ntegrator              = md
dt                      = 0.002
nsteps                  = 50000000
nstxout                 = 50000
nstvout                 = 50000
nstfout                 = 50000
nstcalcenergy           = 100
nstenergy               = 1000
nstlog                  = 1000
;
cutoff-scheme           = Verlet
nstlist                 = 20
rlist                   = 1.2
vdwtype                 = Cut-off
vdw-modifier            = Force-switch
rvdw_switch             = 1.0
rvdw                    = 1.2
coulombtype             = PME
rcoulomb                = 1.2
;
tcoupl                  = Nose-Hoover
tc_grps                 = MEMB SOLV
tau_t                   = 1.0 1.0
ref_t                   = 298.15 298.15
;
DispCorr                = EnerPres
pcoupl                  = no
;pcoupltype              = semiisotropic
;tau_p                   = 5.0
;compressibility         = 4.5e-5  4.5e-5
;ref_p                   = 1.0     1.0
;
constraints             = h-bonds
constraint_algorithm    = LINCS
continuation            = no
;
gen-vel                 = yes
gen-seed                = -1
gen-temp                = 298.15

nstcomm                 = 100
comm_mode               = linear
comm_grps               = MEMB SOLV
```

Script S1. Gromacs code for running classical MD simulations of a surfactant monolayer. Notes: Pressure coupling is turned off (`pcouple = no`) and DispCorr is turned on (`DispCorr = EnerPres`).

```
; Pull code
pull                     = yes
pull_ncoords             = 1
pull_ngroups             = 2
pull_group1_name         = PULL
pull_group2_name         = REFERENCE
pull_group2_pbcatom      = -1
pull_coord1_type         = umbrella
pull_coord1_geometry     = direction-periodic
pull_coord1_dim          = N N Y #z axis is reaction coordinate
pull-coord1-vec          = 0.0 0.0 -1.0
pull_coord1_groups       = 1 2
pull_coord1_start        = yes
pull_coord1_rate         = 0.01
pull_coord1_k            = 1000
```

Script S2. Pull code sample for Section 3.2 from mdp file.

```
; Pull code
pull                     = yes
pull_ncoords             = 1
pull_ngroups             = 2
pull_group1_name         = PULL
pull_group2_name         = REFERENCE
pull-coord1-type         = external-potential
pull-coord1-potential-provider = awh
pull-coord1-geometry     = direction
pull-group2-pbcatom      = -1
pull_coord1_dim          = N N Y #z axis is rxn coordinate
pull-coord1-vec           = 0.0 0.0 -1.0
pull_coord1_groups     = 1 2

; AWH code
awh                      = yes
awh-nbias                = 1
awh1-ndim                = 1
awh1-dim1-coord-index    = 1
awh1-dim1-start          = 2
awh1-dim1-end            = 8.4
awh1-dim1-force-constant = 180000
awh1-dim1-diffusion      = 5e-5 ;nm2/ps
awh1-error-init          = 30 ;kJ/mol
awh1-share-multisim      = yes
awh1-share-group         = 1
awh1-equilibrate-histogram = yes
```

Script S3. AWH code sample for Section 3.4 from mdp file.

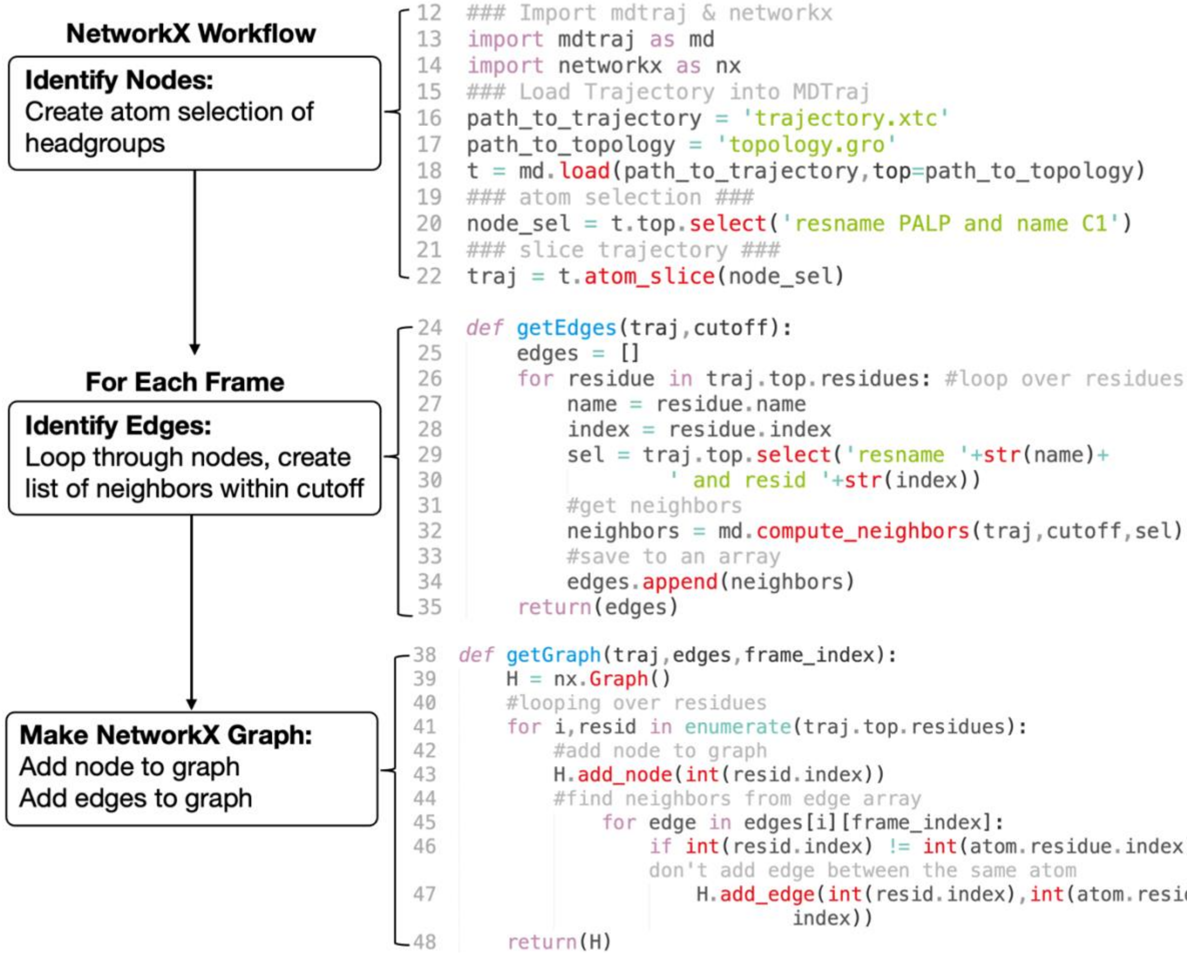


```
### Import mdtraj & networkx
import mdtraj as md
import networkx as nx
### Load Trajectory into MDTraj
path_to_trajectory = 'trajectory.xtc'
path_to_topology = 'topology.gro'
t = md.load(path_to_trajectory,top=path_to_topology)
### atom selection ###
node_sel = t.top.select('resname PALP and name C1')
### slice trajectory ###
traj = t.atom_slice(node_sel)

def getEdges(traj,cutoff):
    edges = []
    for residue in traj.top.residues: #loop over residues
        name = residue.name
        index = residue.index
        sel = traj.top.select('resname '+str(name)+
                ' and resid '+str(index))
        #get neighbors
        neighbors = md.compute_neighbors(traj,cutoff,sel)
        #save to an array
        edges.append(neighbors)
    return(edges)

def getGraph(traj,edges,frame_index):
    H = nx.Graph()
    #looping over residues
    for i,resid in enumerate(traj.top.residues):
        #add node to graph
        H.add_node(int(resid.index))
        #find neighbors from edge array
            for edge in edges[i][frame_index]:
                if int(resid.index) != int(atom.residue.index): #
                don't add edge between the same atom
                    H.add_edge(int(resid.index),int(atom.residue.
                            index))
    return(H)
```

Script S4. Workflow and code for creating a NetworkX graph from an MD trajectory. Example code for FA headgroup selection, where the headgroups are C1 carbons from residue with residue name "PALP". The input trajectories for `getEdges()` and `getGraph()` must already be sliced by lipid type. Note that this code sample has been simplified from its original version and should be considered pseudocode representing the workflow.

4 Calculations

The complex dissolution equilibria of MCFAs make it difficult to determine the contribution of MCFAs to the surface pressure of a mixture. However, analytical methods can be used to estimate the retention from surface pressure-area (Π-A) isotherms. To determine the individual contribution of lauric acid to a mixture of fatty acids, the isotherm of a quaternary mixture (Proxy) is compared to that of a tertiary mixture (Control), with lauric acid absent from the latter. The calculated concentration of the Proxy is then analytically altered by removing the mole fraction contribution of lauric acid by the equation

$$[Mix] = \frac{2n_{MA}+4n_{PA}+3n_{SA}}{V_{LA}+V_{MA}+V_{PA}+V_{SA}}, \tag{4.1}$$

where n and V correspond to moles and volume of each species, respectively. Because Π-A isotherms are surface-specific, removing the mole fraction of the lauric acid contribution will shift the area per lipid (APL)

such that the isotherm will align with that of the tertiary mixture if lauric acid is completely absent from the interface. We can then quantify the retention of lauric acid by the equation

$$\frac{APL_{corrected} - APL_{control}}{APL_{corrected} - APL_{uncorrected}} \times 100\% = \%LA\ retained, \tag{4.2}$$

where the APLs are extracted from the liquid condensed (LC) phase. Plots of the resulting P-A isotherms with the corrected and uncorrected traces are provided below. Results correspond to 50%, -10%, and 30% LA retention for pH 2, 5.6, and 8.2, respectively.

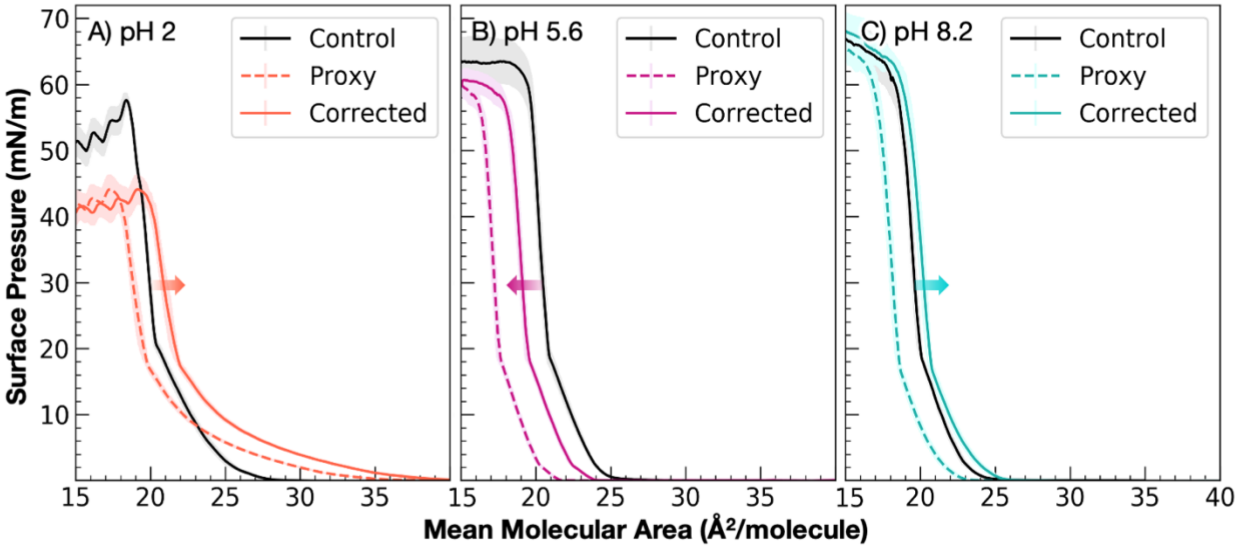

Supplement: Supplementary file 1 — jp4c03335_si_001.pdf [file jp4c03335_si_001.pdf]
